# Miscellaneous Notices

**Published:** 1843-01

**Authors:** 


### MISCELLANEOUS NOTICES.

*Medical News and Library.*—A new candidate for public patronage, under this title, has just been started by Messrs. Lea & Blanchard, of Philadelphia. It is to be published monthly, and in addition to medical news, will contain series of Lectures on the principal branches of medical science. These lectures will have separate paging and constitute "a student's library."

*Medical Examiner.*—This journal has been changed from a weekly to a semi-monthly, and will be hereafter conducted by Dr. Clymer. Arrangements have been made for publishing "a regular series of Lectures by the prominent clinical professors in Paris."

Wilson & Co., of New York, the publishers of what is called (*par excellence?*) "cheap literature," have undertaken the republication of the *London Lancet.* The Lancet is edited (and was originally established) by Mr. Wakely, a member of the British House of Commons; it is of twenty or more year's standing, and is in many respects a valuable periodical.

Dr. Reynell Coates, of Philadelphia, has started a literary and scientific journal entitled the "*Literary Age.*" Dr. Coates is favorably known, both in and out of the profession, as a polished and facile writer; we heartily wish success to his new enterprise. C.

